# Downregulated Expression of CLEC9A as Novel Biomarkers for Lung Adenocarcinoma

**DOI:** 10.3389/fonc.2021.682814

**Published:** 2021-09-20

**Authors:** Fang Miao, Zhiguo Lou, Shuhua Ji, Dan Wang, Yaolan Sun, Huan Liu, Chenggang Yang

**Affiliations:** ^1^School of Basic Medical Sciences, Shandong First Medical University, Jinan, China; ^2^Department of General Education, Shandong First Medical University, Jinan, China; ^3^Department of BigData, Beijing Medintell Bioinformatic Technology Co., LTD, Beijing, China; ^4^Department of Research and Development, Gu’an Bojian Bio-Technology Co., LTD, Langfang, China

**Keywords:** CLEC9A, lung adenocarcinoma, functional network analysis, cell proliferation, biomarkers

## Abstract

**Purpose:**

Abnormal CLEC9A expression is concerned with carcinogenesis. However, the role of CLEC9A in lung adenocarcinoma (LUAD) remains unknown. The goal of this study was to reveal the role of CLEC9A in LUAD based on bioinformatics and cellular functional experiments.

**Materials and methods:**

Data available from The Cancer Genome Atlas (TCGA) were employed to study CLEC9A expression and mutations in LUAD. Expression and alterations of CLEC9A were analyzed using UALCAN and cBioPortal, respectively. Kaplan–Meier analysis was used to analyze the effect of CLEC9A on the survival of LUAD. Protein–protein interaction (PPI) network was built using GeneMANIA analysis. The similar genes of CLEC9A were obtained using GEPIA analysis, while co-expression genes correlated with CLEC9A were identified using LinkedOmics analysis. The effects of CLEC9A expression on immune cell infiltration was assessed. The effect of CLEC9A on the proliferation, apoptosis, cell cycle distribution, and invasion of human LUAD cells was detected in the LUAD cell line.

**Results:**

CLEC9A was downregulated and the CLEC9A gene was often altered in LUAD. The survival of LUAD patients was correlated with the expression level of CLEC9A. The similar genes of CLEC9A were linked to functional networks involving positive regulation of interleukin-12 production, plasma membrane and CD40 receptor binding, primary immunodeficiency, intestinal immune network for IgA production, and cell adhesion molecules pathways. Cell cycle, apoptosis, EMT, and RAS/MAPK were significantly enriched pathways in positive and negative correlation genes with CLEC9A. A difference in the immune infiltration level of immune cell between the high and low CLEC9A expression groups was observed. Somatic cell copy number alternations (CNAs) of the CLEC9A, including arm-level gain and arm-level deletion, observably changed the infiltration levels of B cells, CD4+ T cells, macrophages, and neutrophils in LUAD. Except for LAG3, the expression of CD274, CTLA4, PDCD1, and TIGIT was positively correlated with the expression level of CLEC9A. After transfection, overexpression and knockdown of CLEC9A could affect the proliferation, apoptosis, cell cycle distribution, and invasion of LUAD cells.

**Conclusion:**

CLEC9A is associated with prognosis and tumor immune microenvironment of LUAD, suggesting that CLEC9A may be considered as a novel biomarker for LUAD.

## Introduction

Lung cancer remains the most frequent cause of malignancy-related mortality worldwide, due to its unsatisfactory cure rate, high metastatic rate, and poor prognosis (1–3). It is now generally believed that cancer, especially lung cancer, is the result of interactions between environmental and genetic factors (4, 5). The most common type of lung cancer is non-small cell lung cancer. Lung adenocarcinoma (LUAD), the major type of non-small cell lung cancer, is considered to be a multistep process of carcinogenesis (6). Despite improvements in therapeutic modalities, the 5-year overall survival for LUAD is still low. Thence, it is urgent to identify new robust biomarkers and therapeutic target to assist the treatment of LUAD.

The study of human diseases ultimately depends on understanding the genome and its functions (7). Rapid development of bioinformatics has provided novel tools to research cancer in a more comprehensive fashion (8). Many researchers study the cancer mechanisms and search for robust biomarkers by integrating omics data from different platforms (9–11). Based on the TCGA dataset, Feng et al. have shown that patients with LUAD have a high HMGB1 level, which indicates poor overall survival, suggesting that these novel developed genomic sample databases and methods can find biomarkers for LUAD (12). Some molecular biomarkers have been studied to be involved in the pathogenesis of lung cancer and have clinical associations with LUAD (13–15). With the help of bioinformatics, we identified new reliable biomarkers to understand the underlying mechanisms of LUAD.

Here, we analyzed CLEC9A expression and mutations in data from patients with LUAD in TCGA. Based on multi-dimensional analysis, we analyzed genomic alterations and functional networks related to CLEC9A in LUAD. The effects of CLEC9A expression on immune cell infiltration was assessed. The impact of CLEC9A on the proliferation, apoptosis, cell cycle distribution, and invasion of human LUAD cells was also detected. Therefore, our findings could potentially find novel targets for LUAD treatment.

## Materials and Methods

### CTD Analysis

The Comparative Toxicogenomics Database (CTD; http://ctdbase.org/) is a premier public database for uncovering the mechanisms by which chemical exposure affects human health functions (16). We used CTD to find the correlation between CLEC9A and tumor.

### UALCAN Analysis

UALCAN (http://ualcan.path.uab) is a premier public resource to further explore TCGA gene expression data (9). UALCAN allows analysis of relative expression across cancer and normal samples, as well as indifferent cancer subgroups based on individual cancer clinicopathological information (17). We utilized UALCAN to perform the expression of CLEC9A in LUAD.

### c-BioPortal Analysis

The cBio Cancer Genomics Portal (http://cbioportal.org) is a web interface for revealing and evaluating multidimensional cancer genomics datasets, currently including 225 cancer studies (18). The CLEC9A gene mutation and expression information in LUAD were obtained based on the cBioPortal’s online instructions.

### Kaplan–Meier Analysis

The mRNA expression data LUAD patient sample and clinical information were downloaded from UCSC Xena (https://gdc.xenahubs.net). The following samples were excluded: (1) “0” gene expression value and (2) insufficient survival information. A total of 513 LUAD patients were obtained. According to the median of CLEC9A expression, the data of 513 LUAD patients were divided into a high CLEC9A expression group and a low CLEC9A expression group. Kaplan–Meier analysis was performed to compare the overall survival rate between the high and low CLEC9A expression groups using the *p*-value determined in the log-rank test.

### GeneMANIA Analysis

GeneMANIA (http://www.genemania.org) is a public resource for building protein–protein interaction (PPI) network, producing hypotheses about gene function, displaying gene lists, and prioritizing genes for functional assays (19). We constructed the PPI network of proteins interacting with and associated with CLEC9A using GeneMANIA.

### GEPIA Analysis

GEPIA (Gene Expression Profiling Interactive Analysis; http://gepia.cancer-pku.cn/) is a novel web interface for analyzing the RNA sequencing expression data from the TCGA database on the standard processing pipeline (20). GEPIA was used to obtain similar genes of CLEC9A. The thresholds for similar genes of CLEC9A were defined as Pearson correlation coefficient *r* ≥ 0.5.

### GeneCoDis3 Analysis

Gene Ontology (GO) classification and Kyoto Encyclopedia of Genes and Genomes (KEGG) pathway enrichment analysis were performed using GeneCoDis3 (http://genecodis.cnb.csic.es/analysis). *p*-value < 0.05 was considered as statistically significant.

### LinkedOmics Analysis

The LinkedOmics database (http://www.linkedomics.org/login.php) is a web server for analyzing cancer-associated multi-dimensional datasets of 32 cancer types and 11,158 patients (21). Genes positively and negatively correlated with CLEC9A expression were obtained using LinkedOmics. The screening criteria was *p* value < 0.05 and |*r*| > 0.5. GSCALite (http://bioinfo.life.hust.edu.cn/web/GSCALite/) is a user-friendly web resource for dynamic analysis and visualization of gene set in cancer, which will be of broad utilities to cancer researchers (22). Pathway enrichment analysis of co-expressed gene with CLEC9A was performed using GSCALite.

### The Effects of CLEC9A Expression on Immune Cell Infiltration

The single-sample gene-set enrichment analysis (SSGSEA) algorithm was performed to compare differences in immune cell infiltration between high and low CLEC9A expression groups. Then, the effect of somatic cell copy number alternations (CNAs) of the CLEC9A on immune cell infiltration levels was assessed using the Tumor Immune Estimation Resource (TIMER, https://cistrome.shinyapps.io/timer/) composed of six immune cell types (B cells, CD4+ T cells, CD8+ T cells, neutrophils, macrophages, and dendritic cells). To explore the relationship between immune checkpoints (CD274, CTLA4, LAG3, PDCD1, and TIGIT) and CLEC9A, we evaluated the expression of these immune checkpoints in the high and low CLEC9A expression groups using Wilcoxon test.

### Cell Culture

The human LUAD cell line A549, Calu-3, NCI-H1975, NCI-H1395, and immortalized human bronchial epithelial cell line BEAS-2B were purchased from the American Type Culture Collection (Rockville, MD, USA) and cultured in high-glucose Dulbecco’s Modified Eagle’s Medium (DMEM; Hyclone, Logan, UT, USA) supplemented with 10% fetal bovine serum (FBS; Hyclone, Logan, UT, USA) and 1% penicillin/streptomycin (Sangon; Shanghai, China) at 37°C in 5% CO_2_.

### Construction and Transfection of Plasmids and Small Interfering RNA (siRNA)

The overexpression plasmid (pcDNA3.1-CLEC9A) and the control vector (pcDNA3.1-vector), siRNA-CLEC9A, and non-targeting control siRNA (NC-siRNA) were purchased from GenePharma (Shanghai, China). Cells were seeded into six-well plates, and the media were aspirated once the cells grew to 65%–70% confluence. Cells were transfected with the corresponding plasmid or siRNA with Lipofectamine 2000 (Thermo Fisher Scientific, Waltham, USA) following the manufacturer’s instructions. After 24 h post-transfection, the transfection medium was changed with fresh serum medium. Cells were then cultured for 24 h and collected for further experiments.

### Quantitative Real−Time Polymerase Chain Reaction (RT−qPCR)

Total RNA was extracted from cells using TRIzol reagent (Tiangen, Beijing, China) and subjected to reverse transcription with the Fast Quant RT Kit (Tiangen) following the manufacturer’s protocol. RT-qPCR assay was carried out with the Super Real PreMix Plus SYBR Green (Tiangen) as described on the manufacturer’s instructions. The primers of CLEC9A and GAPDH were listed as follows: CLEC9A, 5′-CAGCCCATAACAGCAGTCCT-3′ (forward) and 5′-CATCCTGAGACAACCCCACC-3′ (reversed) and GAPDH, 5′-GGAGCGAGATCCCTCCAAAAT-3′ (forward) and 5′-GGCTGTTGTCATACTTCTCATGG-3′ (reversed). Relative quantification of CLEC9A levels was assessed by using the 2^−ΔΔCt^ method with normalization to the endogenous control.

### Cell Proliferation Analysis

Cell viability was detected using the MTT assay. In brief, cells were seeded and incubated at a concentration of 3 × 10^3^ in 100 μl of medium into 96-well plates and cultured for 24 h. Then, 20 μl of MTT medium was added into each well for 4 h. The medium was removed, and 150 μl of DMSO was used to dissolve the formazan crystal. The optical density of each well at 570 nm was detected with a SpectraMax M5 plate reader system (Molecular Devices, Sunnyvale, California, USA).

### Apoptosis Analysis

An apoptosis detection kit (Beyotime, Shanghai, China) was applied to detect cell apoptosis according to the manufacturer’s protocols. Briefly, transfected cells were harvested and prepared, and subsequently stained with Annexin V-FITC and propidium iodide (PI) in the dark for 15 min. Finally, apoptotic cells were analyzed immediately by flow cytometer (BD Biosciences, San Jose, CA, USA).

### Cell Cycle Analysis

Transfected cells were harvested, fixed with cold 70% ethanol, and incubated at 4°C overnight. Then, we stained the cells with PI, followed by analysis of cell cycle distribution on a flow cytometer (BD Biosciences). The proportion of the cells was analyzed using CellQuest Pro software version 5.1 (BD Biosciences).

### Cell Invasion Analysis

Cell invasion was detected using transwell assay. Transfected cells resuspended in serum-free medium were placed into the upper chamber of a 24-transwell plate with an 8-µm pore filter (BD Biosciences). Then, 500 µl of growth medium containing 10% FBS was added into the lower chamber. After incubation for 24 h, the cells that moved through the underside of the membrane filter were fixed with 4% paraformaldehyde and stained with 0.25% crystal violet. The number of invaded cells was counted and the images were photographed under a light microscope (Olympus, Tokyo, Japan).

## Results

### CLEC9A Expression in LUAD

**Figure 1:**
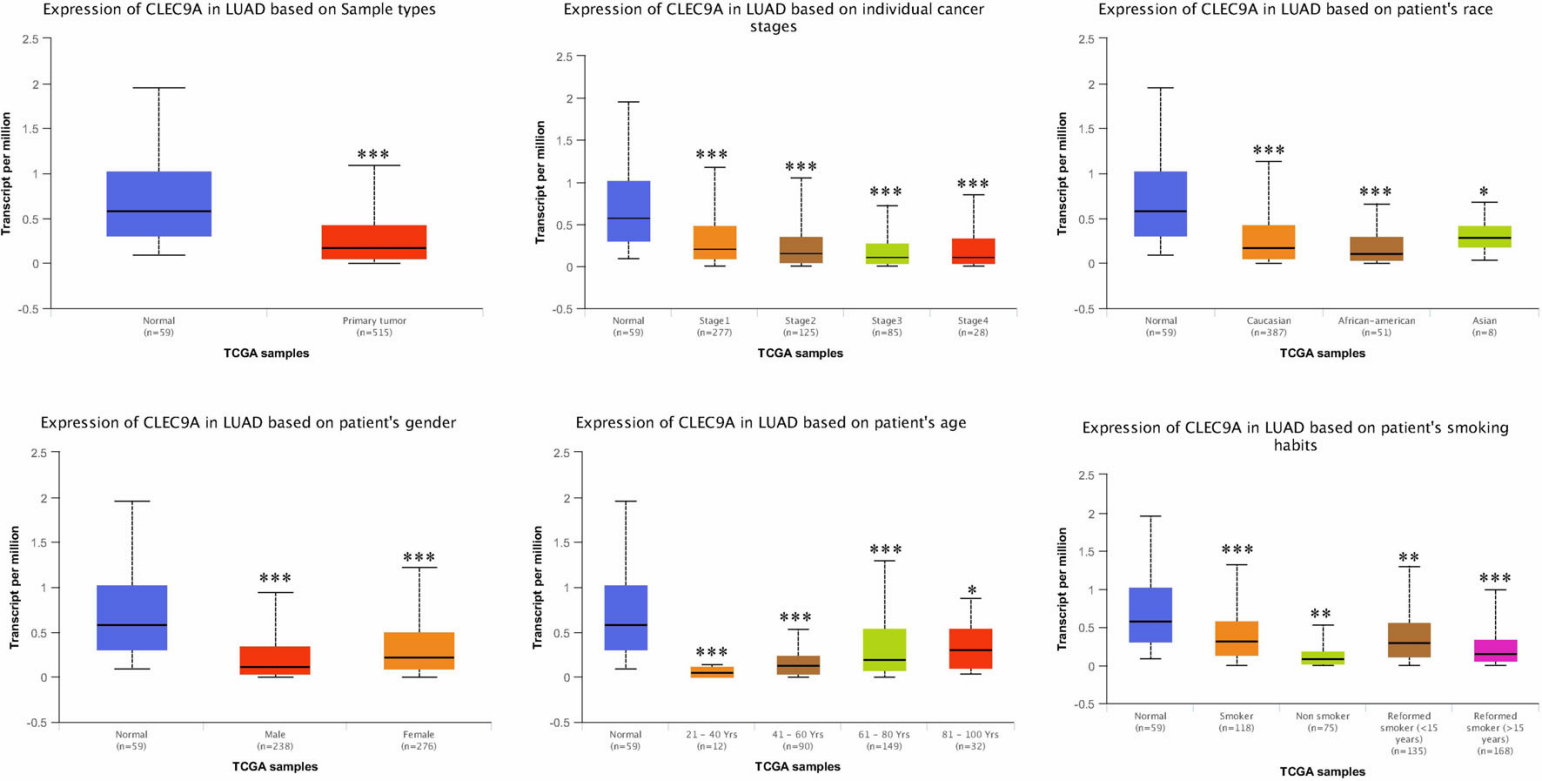
CLEC9A expression in subgroups of patients with LUAD, stratified based on gender, age, and other criteria. *p < 0.05, **p < 0.01, ***p < 0.001.

We initially investigated cancers related to CLEC9A and found that CLEC9A had the highest correlation with lung cancer (**Table 1**). Then, we studied CLEC9A expression levels in multiple LUAD samples from TCGA, and the results displayed that CLEC9A was significantly downregulated in LUAD (**Figure 1**). We also evaluated CLEC9A expression on the basis of multiple clinic pathological features in LUAD samples from TCGA. The expression level of CLEC9A was significantly lower in LUAD patients than the normal control in subgroup analyses based on stage, race, gender, age, and smoking habits (**Figure 1**).

**Table 1:** Cancer associated with CLEC9A.

Disease Name	Disease ID	Inference Score	Reference Count
Lung Neoplasms	MESH:D008175	25.05	27
Cell Transformation, Neoplastic	MESH:D002471	23.75	22
Liver Neoplasms, Experimental	MESH:D008114	22.72	175
Cholangiocarcinoma	MESH:D018281	21.49	9
Liver Neoplasms	MESH:D008113	17.06	101
Skin Neoplasms	MESH:D012878	15.81	10
Ovarian Neoplasms	MESH:D010051	15.53	5
Sarcoma	MESH:D012509	14.43	2
Colonic Neoplasms	MESH:D003110	14.17	4
Neoplasms, Experimental	MESH:D009374	13.85	9

### Genomic Alterations and Survival of CLEC9A in LUAD

**Figure 2:**
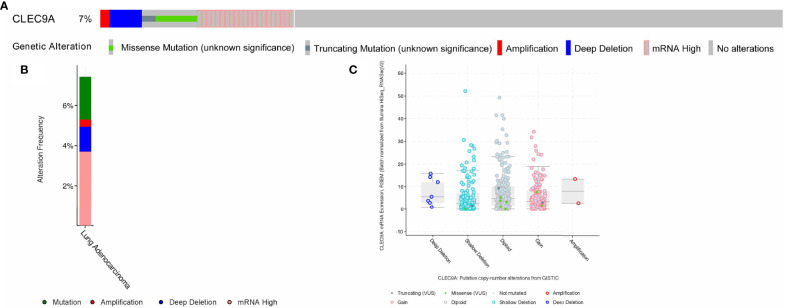
Visual summary of CLEC9A alterations in LUAD. The different types of genetic alterations are highlighted in different colors. **(A)** CLEC9A alterations in LUAD. **(B)** The alteration frequency of CLEC9A in LUAD. **(C)** Expression of CLEC9A in different altered states.

**Figure 3:**
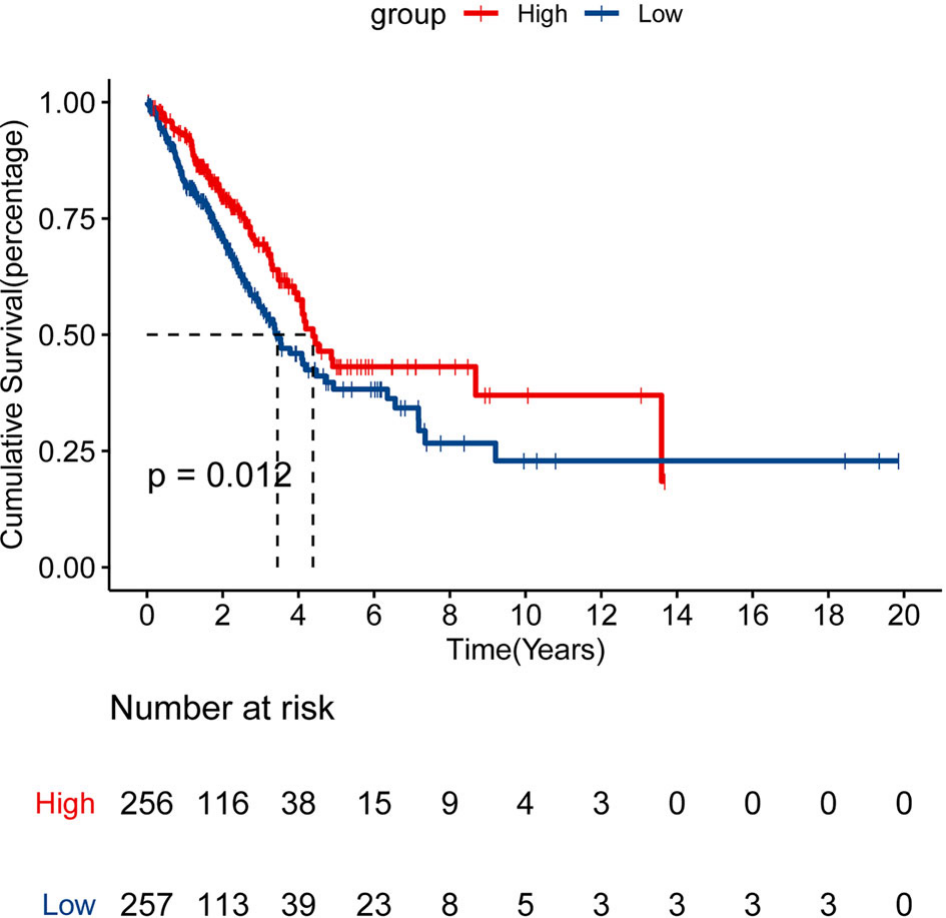
Survival analysis for CLEC9A in LUAD. Kaplan–Meier analysis was performed to compare the overall survival (OS) rate between the high and low CLEC9A expression groups.

We performed the cBioPortal to determine the types and frequency of CLEC9A alterations in LUAD based on sequencing data from the TCGA database. CLEC9A was altered in 42 of 566 (7.42%) LUAD patients (**Figure 2A**). These alterations were mRNA upregulation in 21 cases (3.71%), amplification in 2 cases (0.35%), mutation in 12 cases (2.12%), and deep deletion in 7 cases (1.24%) (**Figures 2B, C**). Thence, mRNA upregulation is the most usual type of CLEC9A DNA copy number variation in LUAD. Based on TCGA data and clinical information, we analyzed the survival curves for patients by comparing LUAD patients with a higher expression CLEC9A to LUAD patients with a lower expression CLEC9A. Kaplan–Meier analysis showed that the overall survival rate of low-expressing CLEC9A was lower than that of high-expressing CLEC9A (*p* = 0.012; **Figure 3**).

### PPI Network Analysis of Interacting Genes With CLEC9A

**Figure 4:**
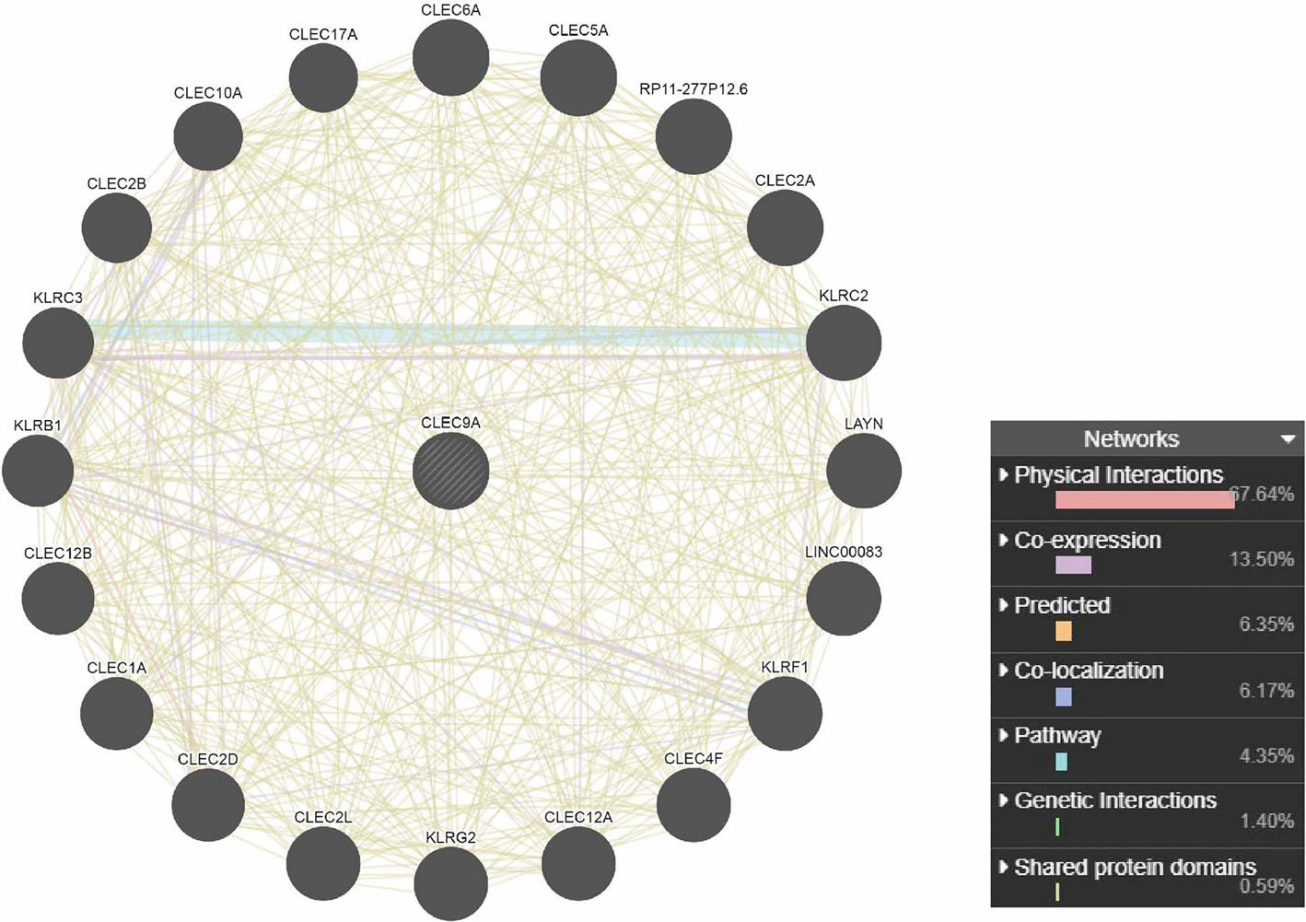
PPI network analysis of interacting genes with CLEC9A. Circles are used to represent nodes, and lines are used to represent edges.

The PPI network analysis of interacting genes with CLEC9A was produced by GeneMANIA. The PPI network consisted of 21 nodes and 447 edges (**Figure 4**). The top five proteins with higher degrees are CLEC17A (degree = 40), CLEC2B (degree = 36), KLRC3 (degree = 34), KLRB1 (degree = 32), and CLEC12B (degree = 30).

### Functional Annotation of Similar Genes of CLEC9A

**Figure 5:**
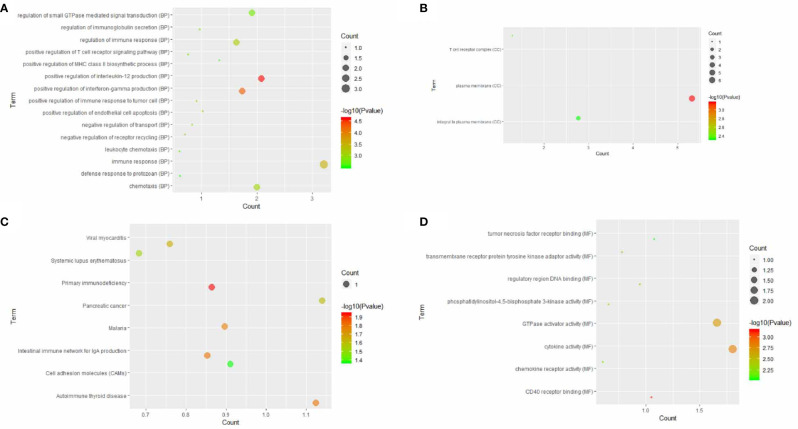
GO terms or KEGG pathways of similar genes of CLEC9A. The *x*-axis shows −log *p*, and the *y*-axis shows GO terms or KEGG pathways. **(A)** Biological process. **(B)** Cellular component. **(C)** Molecular function. **(D)** KEGG pathways.

A total of 15 similar genes of CLEC9A were utilized to perform the GO and KEGG pathway enrichment analysis. Based on GO enrichment analysis, positive regulation of interleukin-12 production (*p* = 2.36E−05), plasma membrane (*p* = 0.000685639), and CD40 receptor binding (*p* = 0.000701477) were markedly enriched GO terms (**Figures 5A–C**). KEGG pathway enrichment analysis showed that primary immunodeficiency (*p* = 0.0115169), intestinal immune network for IgA production (*p* = 0.0153287), and cell adhesion molecules (*p* = 0.0429855) were three significantly enriched pathways (**Figure 5D**).

### Co-Expression Genes Correlated With CLEC9A

**Figure 6:**
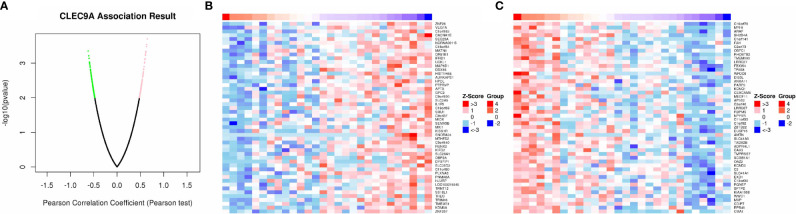
Co-expression genes in correlation with CLEC9A in LUAD. **(A)** A Pearson test was used to analyze correlations between CLEC9A and co-expression genes in LUAD. **(B, C)** Heat maps showing genes positively and negatively correlated with CLEC9A in LUAD. Red indicates positively correlated genes and green indicates negatively correlated genes.

As shown in the volcano plot (**Figure 6A**), 86 genes showed significant positive correlations with CLEC9A, whereas 76 demonstrated significant negative correlations. Hierarchical clustering analysis of 50 significant gene sets positively and negatively correlated with CLEC9A are displayed in **Figures 6B, C**.

### PPI Network Analysis of Co-Expression Genes Correlated With CLEC9A

The PPI network of positive correlation genes with CLEC9A consisted of 88 nodes and 326 edges (**Supplementary Figure 1A**). The PPI network of negative correlation genes with CLEC9A consisted of 78 nodes and 364 edges (**Supplementary Figure 1B**). KEGG pathway analyses of co-expression genes correlated with CLEC9A were performed using GSCALite. Among which, cell cycle, apoptosis, EMT, and RAS/MAPK were significantly enriched pathways in positive and negative correlations genes with CLEC9A (**Supplementary Figure 2**).

### The Effects of CLEC9A Expression on Immune Cell Infiltration

**Figure 7:**
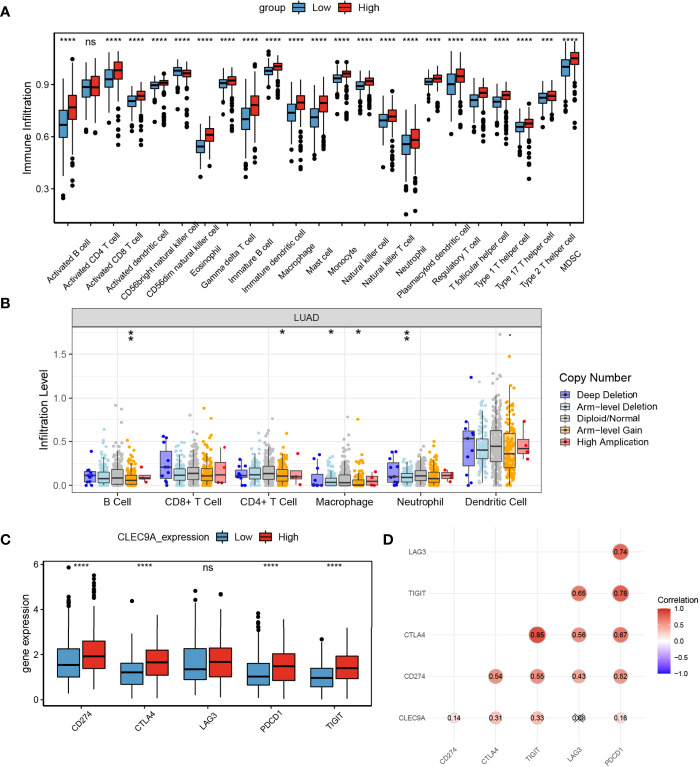
The effects of CLEC9A expression on immune cell infiltration. **(A)** Comparison of immune cell infiltration among high and low CLEC9A expression groups using ssGSEA analysis. **(B)** Effect of the genetic alterations of CLEC9A expression on immune cell infiltration. **(C)** Expression of immune checkpoints between low CLEC9A expression groups. **(D)** The relationship between immune checkpoints and CLEC9A expression. ns, not significant. *p < 0.05, **p < 0.01, ***p < 0.001, ****p < 0.0001.

The differential of immune cell infiltration between high and low CLEC9A expression groups was detected using SSGSEA algorithm. The distribution of immune infiltration cells between high and low CLEC9A expression groups was demonstrated in **Figure 7A**. These results showed that, except for activated CD4 T cells, the immune infiltration level of 22 other cells was different between the high and low CLEC9A expression groups. Next, we studied the effect of changes in CNAs of CLEC9A on immune cell infiltration. As shown in **Figure 7B**, the CNAs of the CLEC9A, including arm-level gain and arm-level deletion, observably changed the infiltration levels of B cells, CD4+ T cells, macrophages, and neutrophils in LUAD. To reveal the relationship between immune checkpoints (CD274, CTLA4, LAG3, PDCD1, and TIGIT) and CLEC9A, we evaluated the expression of these immune checkpoints between high and low CLEC9A expression groups. Except for LAG3, the expression of CD274, CTLA4, PDCD1, and TIGIT was positively correlated with the expression level of CLEC9A (**Figures 7C, D**). The above results show that CLEC9A may be involved in the regulation of tumor immune microenvironment in LUAD patients.

### Impact of CLEC9A on the Proliferation, Apoptosis, Cell Cycle Distribution, and Invasion of Human LUAD Cells

To further uncover the confirmed effect of CLEC9A in LUAD, we adopted A549 and BEAS-2B to CLEC9A transfection. RT-qPCR results showed that the expression of CLEC9A was markedly increased in the CLEC9A group than in the empty vector group and the control group (**Supplementary Figure 3A**). These results showed that the CLEC9A was successfully transfected into A549 and BEAS-2B cells. Subsequently, MTT assay was used to detect the effect of CLEC9A on cell proliferation. CLEC9A overexpression remarkably inhibited cell proliferation of A549 cells, while CLEC9A overexpression had no significant effect on the cell proliferation of BEAS-2B cells (**Supplementary Figure 3B**). In conclusion, these results suggest that CLEC9A only affects the proliferation of LUAD cells and has no effect on the proliferation of normal lung cells, which indicates that it is necessary to further study the biological role of CLEC9A in LUAD cells.

**Figure 8:**
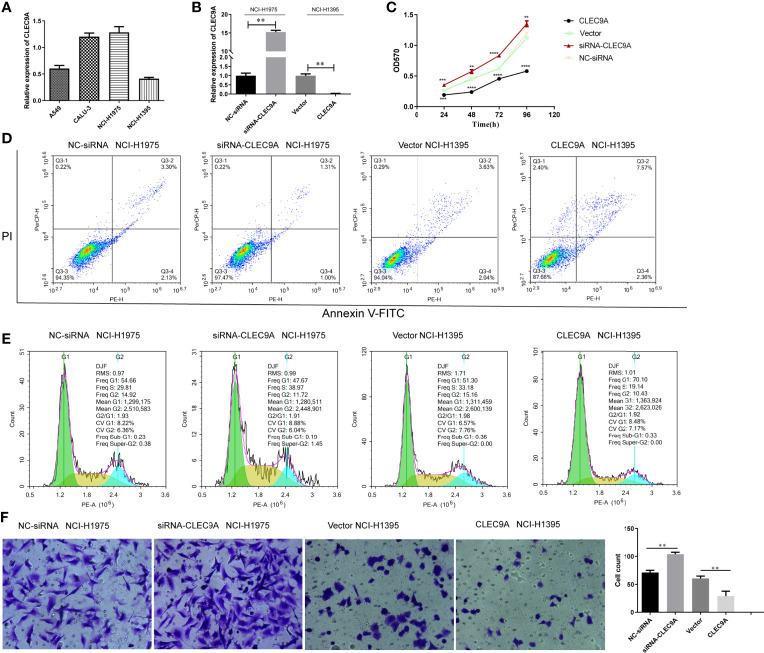
Impact of CLEC9A on the proliferation, apoptosis, cell cycle distribution, and invasion of human LUAD cell lines. **(A)** CLEC9A expression level in A549, Calu-3, NCI-H1975, and NCI-H1395 cells was detected by RT-qPCR. **(B)** Transfection efficacy in NCI-H1975 and NCI-H1395 cells was detected by RT-qPCR. **(C)** The impact of CLEC9A expression on the proliferation was determined by MTT assay. **(D)** Cell apoptosis ratio was measured by using the Annexin V-FITC/PI double staining. **(E)** Cell cycle distribution was evaluated by using flow cytometric analyses. **(F)** Cell invasion was performed by using transwell assay. **p < 0.01, ***p < 0.001, ****p < 0.0001.

We then studied the expression of CLEC9A by RT-qPCR in various human LUAD cell lines, including A549, Calu-3, NCI-H1975, and NCI-H1395. Among the cell lines examined, the expression of CLEC9A in NCI-H1395 cells was the lowest, while that in NCI-H1975 cells was the highest (**Figure 8A**). To control the endogenous expression of CLEC9A in NCI-H1395 and NCI-H1975 cells, pcDNA3.1-CLEC9A construct or CLEC9A siRNA was used, respectively. The expression of CLEC9A increased significantly upon transfection with pcDNA3.1-CLEC9A in NCI-H1395 cells and it decreased upon transfection with CLEC9A siRNA in NCI-H1975 cells (**Figure 8B**).

To uncover the functional role of CLEC9A in LUAD cell proliferation, MTT assays were carried out. The cell proliferation was significantly increased in the CLEC9A siRNA transfected NCI-H1975 cells compared to that in the NC-siRNA transfected cells (**Figure 8C**). In contrast, cell proliferation observably decreased in the pcDNA3.1-CLEC9A transfected NCI-H1395 cells, compared to that in the control vector transfected cells (**Figure 8C**). Next, we used flow cytometric analyses to evaluate the effect of CLEC9A expression on apoptosis and cell cycle distribution. The rate of apoptosis was decreased in cells transfected with the CLEC9A siRNA transfected NCI-H1975 cells compared to that in the NC-siRNA transfected cells (**Figure 8D**). The rate of apoptosis was increased in NCI-H1395 cells after the overexpression of CLEC9A (**Figure 8D**). CLEC9A knockdown decreased the proportion of cell cycle arrest in the G1 phases in NCI-H1975 cells, while CLEC9A overexpression increased the cell cycle arrest in NCI-H1395 cells (**Figure 8E**). Furthermore, we performed transwell assay to illustrate the effect of CLEC9A expression on cell invasion. The number of invading CLEC9A siRNA transfected NCI-H1975 cells was markedly increased compared to that of NC-siRNA transfected cells (**Figure 8F**). In contrast, the number of invading pcDNA3.1-CLEC9A NCI-H1395 cells was significantly decreased relative to that of control vector transfected cells (**Figure 8F**). To sum up, overexpression and knockdown of CLEC9A can affect the proliferation, apoptosis, cell cycle distribution, and invasion of LUAD cells.

## Discussion

C-type lectin domain containing 9A (CLEC9A), also called DNGR-1, is located in the “Dectin-1 cluster” of related receptors, which is encoded within the natural killer gene complex. Dendritic cells have strong antigen-presenting capabilities and have long been recognized as a key factor in anti-tumor immunity (23). CLEC9A is a potential target to dendritic cell vaccines for anti-tumor use (24). Dendritic cells targeting antigens *via* CLEC9A may enhance anti-tumor immunity (24, 25). CLEC9A is a dendritic cell restricted marker that can detect damaged cells and antigens, so targeting CLEC9A + dendritic cells can promote humoral and cellular immunity (24). In order to understand the potential function of CLEC9A in LUAD and its regulatory network in more detail, we performed bioinformatics analysis of public sequencing data and *in vitro* functional experiments to guide future studies of LUAD.

Lung cancer is the most common cause of cancer-related mortality worldwide, causing more than 1 million deaths each year, and adenocarcinoma is the most common histological type. Recently, despite the continuous progress and development in the treatment of LUAD, the prognosis is still poor and the survival rate over 5 years is low (6, 26). LUAD occurrence and metastasis are relatively usual, even with the tumor diagnosed at an early stage (27). Thence, it is essential to seek new biomarkers for LUAD treatment. Here, we analyzed CLEC9A expression and mutations in data from patients with LUAD in TCGA databases. Using multi-dimensional analysis methods, we performed functional networks related to CLEC9A in LUAD.

DNA copy number variations may have significant genomic significance, interfering with genes and altering genetic content, leading to phenotypic differences (28). Here, we reported that the copy number of CLEC9A was upregulated in LUAD, and that the major type of CLEC9A alteration was mRNA upregulation. Altered CLEC9A expression in LUAD may result from alterations in chromosomal structure. Since CLEC9A plays several important physiological functions, its alteration may cause change in various downstream signaling pathways. GO enrichment analysis of similar genes of CLEC9A are involved in positive regulation of interleukin-12 production and CD40 receptor binding, which is closely related to immunity. The KEGG pathway enrichment analysis results displayed that primary immunodeficiency, Intestinal immune network for IgA production, and Cell adhesion molecules were three significantly enriched pathways.

Based on KEGG pathway analyses of co-expression genes correlated with CLEC9A, cell cycle, apoptosis, EMT, and RAS/MAPK were significantly enriched pathways in positive and negative correlation genes with CLEC9A. Cell cycle pathway controls a kind of cell cycle genes and is a potential treatment regulator for tumor therapy. Disorder of cell cycle is a pivotal indicator of lung cancer (29). The upregulation of HOTAIR was related to resistance of gefitinib through the regulating cell cycle in lung cancer (29). The downregulation of Rac3 markedly induced cell growth inhibition, cell cycle arrest, and apoptosis of LUAD cell lines, which is accompanied by significant downregulation of the cell cycle pathway involved in the tumorigenesis of LUAD (30). Ketamine induces apoptosis in LUAD cells *via* the expression of CD69 (31). PDIA6 induces apoptosis of lung cancer cells by regulating the MAP4K1/JNK signaling pathway (32). EMT has been shown to play a pivotal role in the kinds of cancer, including LUAD. Body of evidence has displayed that activation of EMT signaling promotes lung cancer metastasis and therapeutic resistance (33). Therefore, we speculated that CLEC9A might play pivotal roles in LUAD by regulating the signaling pathway of cell cycle, apoptosis, EMT, and RAS/MAPK. Further studies should verify this hypothesis.

CLEC9A is reported to be a significant target for cancer immunotherapy (24). Therefore, we studied the effect of CLEC9A on tumor immune microenvironment in LUAD. SSGSEA algorithm results showed that, except for activated CD4 T cell, the immune infiltration level of 22 other cells was different between the high and low CLEC9A expression groups. Next, we studied the effect of changes in CNAs of CLEC9A on immune cell infiltration. The CNAs of the CLEC9A, including arm-level gain and arm-level deletion, observably changed the infiltration levels of B cells, CD4+ T cells, macrophages, and neutrophils in LUAD. Except for LAG3, the expression of other immune checkpoints (CD274, CTLA4, PDCD1, and TIGIT) was positively correlated with the expression level of CLEC9A. This finding showed that CLEC9A might be involved in the regulation of tumor immune microenvironment in LUAD patients.

As far as we know, the function of CLEC9A in LUAD cells has not been reported. To further uncover the biological role of CLEC9A, we overexpressed CLEC9A in NCI-H1395 cells with low CLEC9A expression and knocked down CLEC9A in NCI-H1975 cells with high CLEC9A expression. The expression of CLEC9A increased significantly upon transfection with pcDNA3.1-CLEC9A in NCI-H1395 cells and it decreased upon transfection with CLEC9A siRNA in NCI-H1975 cells. Molecular biological experiments were performed to explore the effect of CLEC9A on proliferation, apoptosis, cell cycle distribution, and invasion of human LUAD cells. Our results demonstrated that knockdown of CLEC9A promoted cell proliferation and invasion and suppressed apoptosis and cell cycle arrest in NCI-H1975 cells, and overexpression of CLEC9A suppressed cell proliferation and invasion and promoted apoptosis and cell cycle arrest in NCI-H1395 cells. These data suggested that CLEC9A acts as a regulatory factor in LUAD.

## Conclusion

CLEC9A was markedly downregulated in LUAD and associated with prognosis and tumor immune microenvironment of LUAD. Overexpression and knockdown of CLEC9A could affect the proliferation, apoptosis, cell cycle distribution, and invasion of LUAD cells. Thence, CLEC9A may be a potential target for the treatment of LUAD in the future. Some limitations should be acknowledged in this study; our results were not verified by the results in LUAD clinical samples. This study is a pilot study and further experiments are needed to uncover the pathogenesis of CLEC9A in the LUAD.

## Data Availability Statement

The raw data supporting the conclusions of this article will be made available by the authors, without undue reservation.

## Author Contributions

CY, FM, and ZL contributed to the conception of the study. SJ, DW, YS, and HL contributed materials and performed the experiment. SJ, DW, YS, and HL performed the data analyses. CY, FM, and ZL contributed significantly in writing the manuscript. All authors contributed to the article and approved the submitted version.

## Conflict of Interest

Authors SJ, DW, YS, and HL were employed by the company Beijing Medintell Bioinformatic Technology Co., LTD, Beijing, China. Author CY was employed by the company Beijing Medintell Bioinformatic Technology Co., LTD, Beijing, China and Gu’an Bojian Bio-Technology Co., LTD, Langfang, China.

The remaining authors declare that the research was conducted in the absence of any commercial or financial relationships that could be construed as a potential conflict of interest.

## Publisher’s Note

All claims expressed in this article are solely those of the authors and do not necessarily represent those of their affiliated organizations, or those of the publisher, the editors and the reviewers. Any product that may be evaluated in this article, or claim that may be made by its manufacturer, is not guaranteed or endorsed by the publisher.

## Supplementary Material

Supplementary Figure 1 PPI network of correlations genes with CLEC9A in LUAD. **(A)** Positive correlations genes. **(B)** Negative correlations genes.

Supplementary Figure 2 KEGG pathway of correlations genes with CLEC9A in LUAD. **(A)** Positive correlations genes. **(B)** Negative correlations genes.

Supplementary Figure 3 Overexpression of CLEC9A inhibits LUAD cell proliferation. **(A)** Transfection efficacy were detected by RT-qPCR. **(B)** MTT assay revealed the viability of LUAD cells was suppressed by overexpression of CLEC9A. *P < 0.05, **P < 0.01.
