# Supplementary figures and images for: Downregulated Expression of CLEC9A as Novel Biomarkers for Lung Adenocarcinoma
Source: Front Oncol. 2021 Sep 20;11:682814. doi: 10.3389/fonc.2021.682814 (PMC8489846; doi:10.3389/fonc.2021.682814)

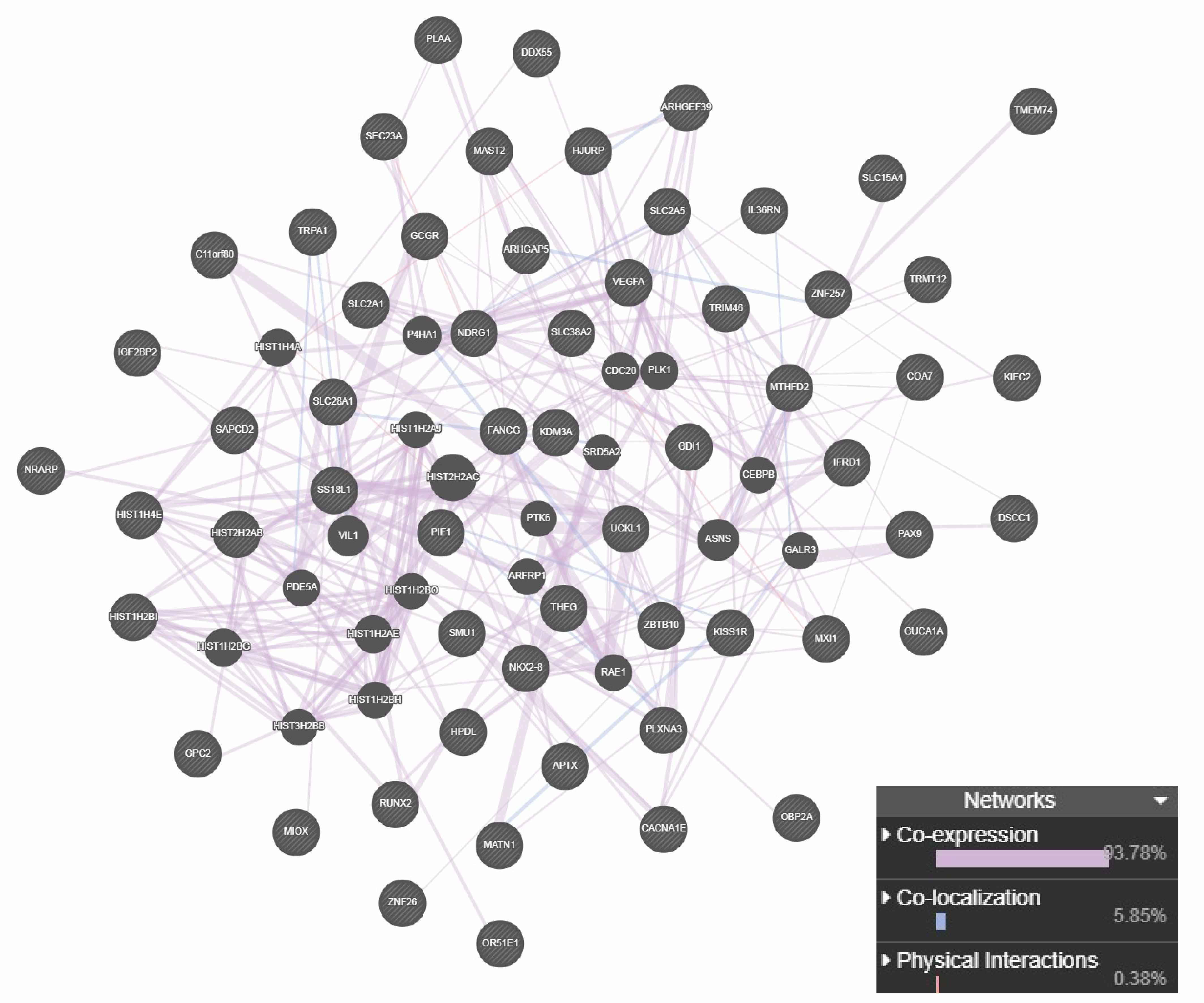

Supplement: Supplementary Figure 1 — PPI network of correlations genes with CLEC9A in LUAD. (A) Positive correlations genes. (B) Negative correlations genes. [file Image_1.jpeg]

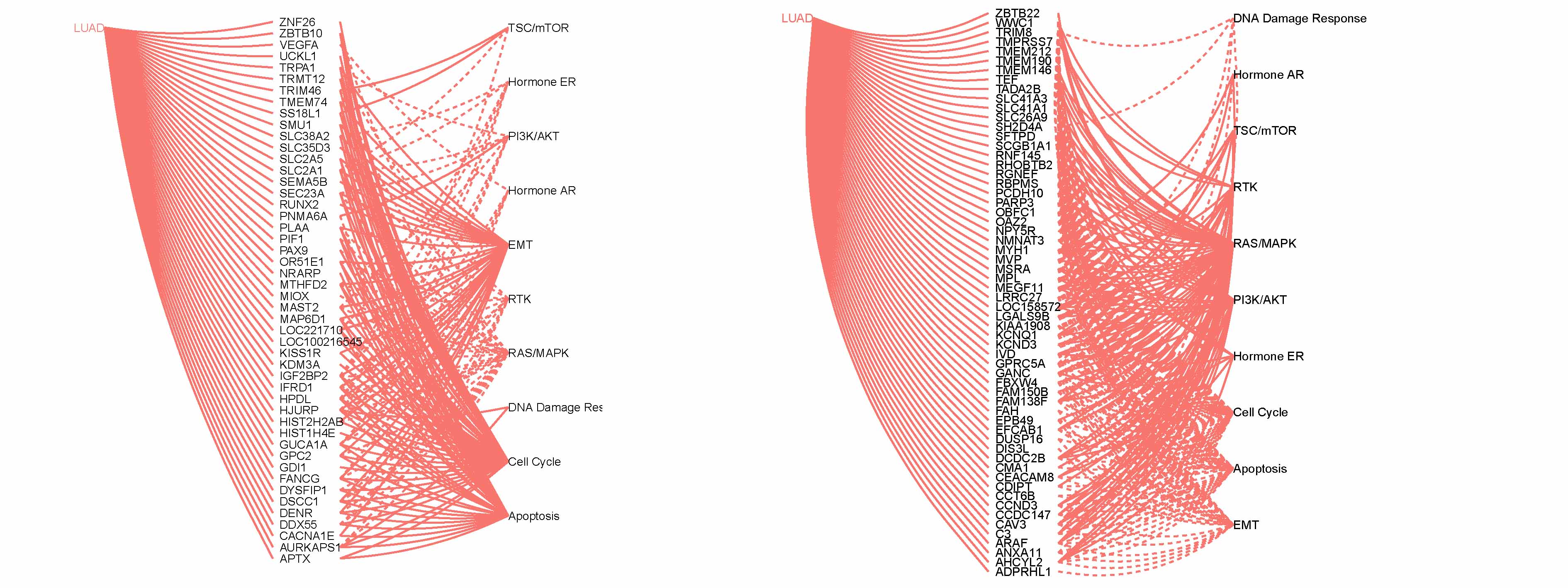

Supplement: Supplementary Figure 2 — KEGG pathway of correlations genes with CLEC9A in LUAD. (A) Positive correlations genes. (B) Negative correlations genes. [file Image_3.jpeg]

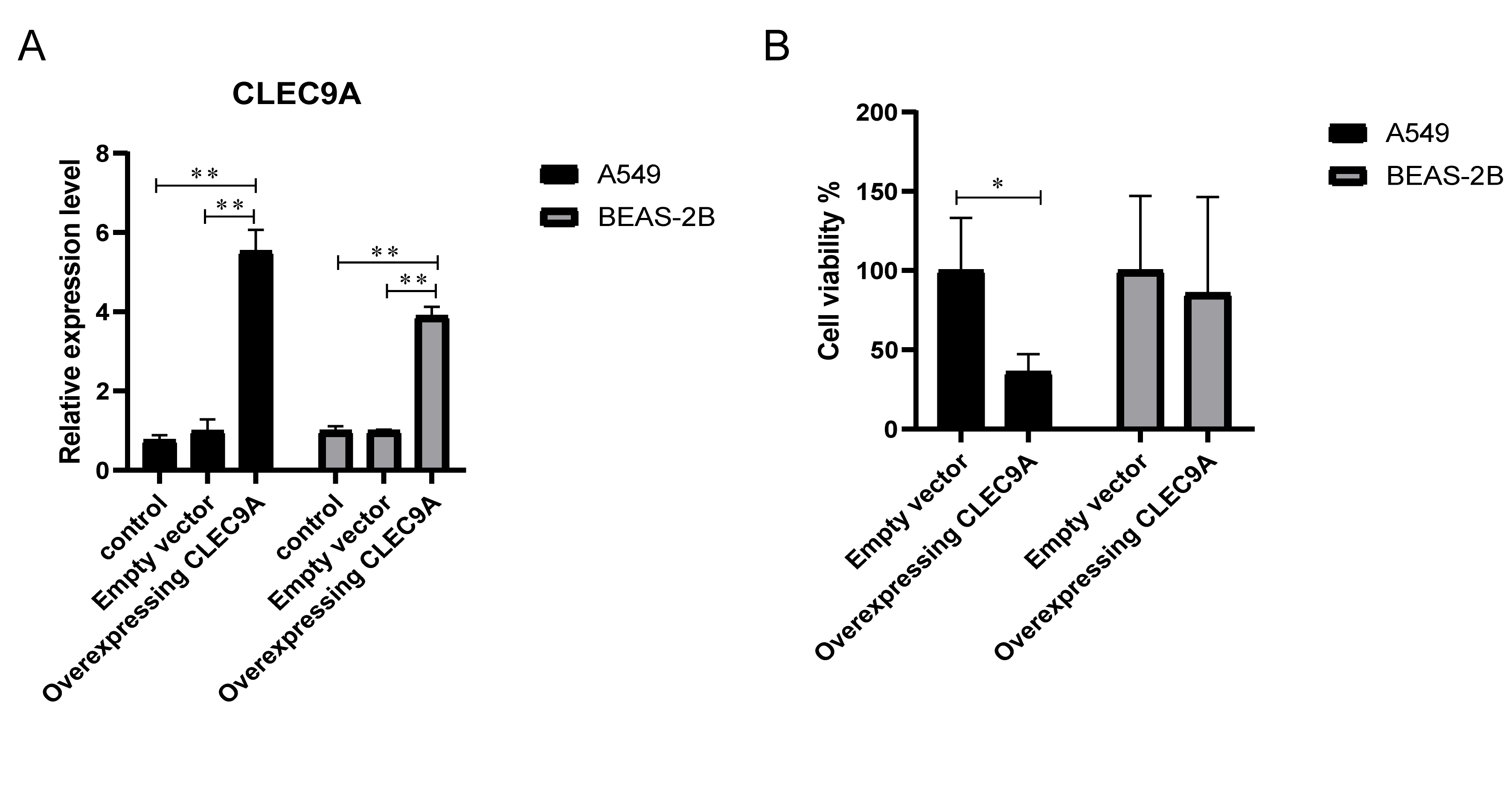

Supplement: Supplementary Figure 3 — Overexpression of CLEC9A inhibits LUAD cell proliferation. (A) Transfection efficacy were detected by RT-qPCR. (B) MTT assay revealed the viability of LUAD cells was suppressed by overexpression of CLEC9A. *P < 0.05, **P < 0.01. [file Image_4.tif]

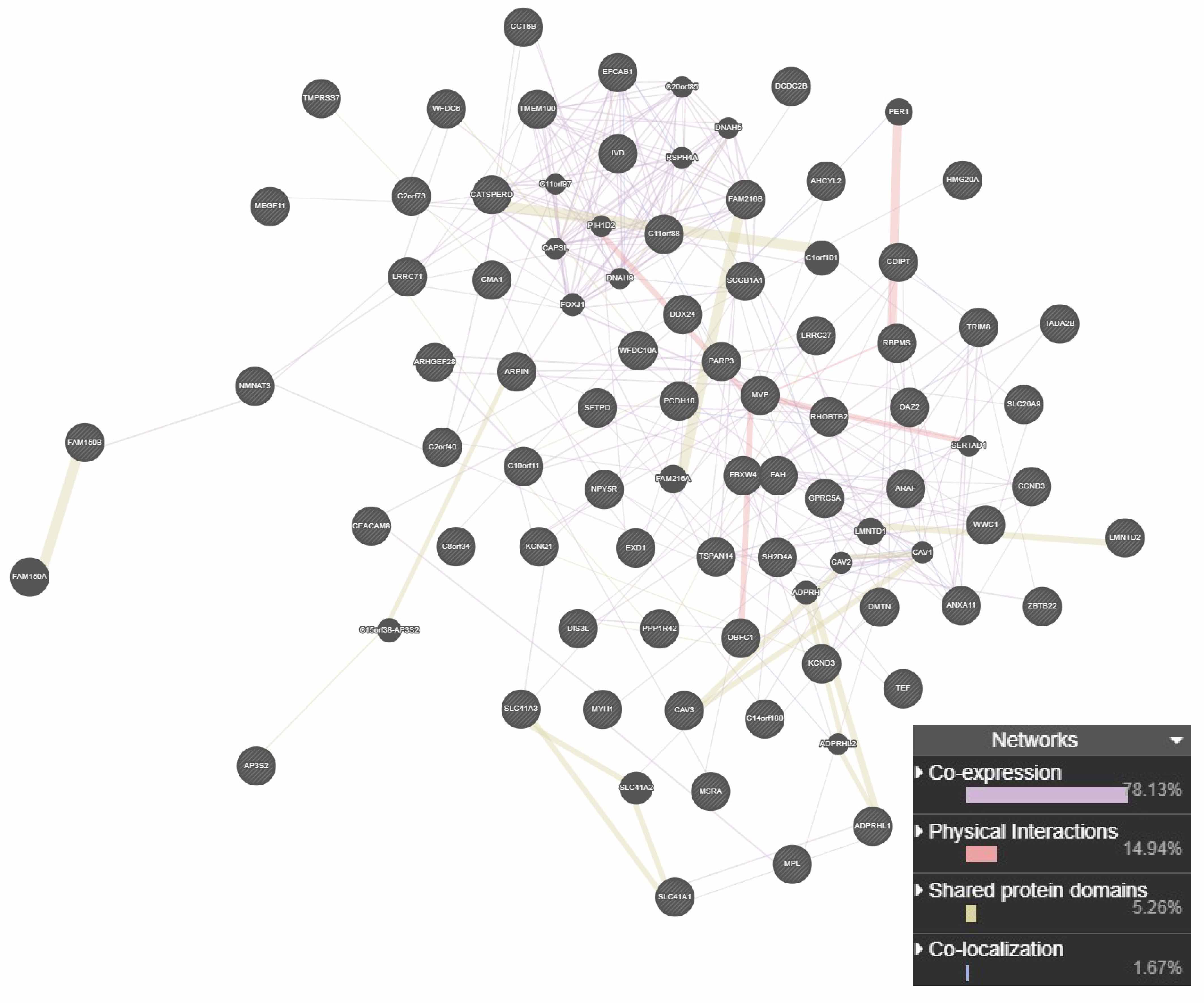

Supplement: Supplementary file 4 [file Image_2.jpeg]
